# Insulin-like peptide 3 is not a biomarker for pancreatic ductal adenocarcinoma

**DOI:** 10.3389/fendo.2025.1625906

**Published:** 2025-08-27

**Authors:** Ravinder Anand-Ivell, Xinyuan Yang, Ruediger Braun, Hendrik Ungefroren, Richard Ivell

**Affiliations:** ^1^ School of Biosciences, University of Nottingham, Sutton Bonington, United Kingdom; ^2^ Department of Surgery, University Medical Center Schleswig-Holstein (UKSH), Lübeck, Germany; ^3^ First Department of Medicine, University Medical Center Schleswig-Holstein (UKSH), Lübeck, Germany; ^4^ School of Veterinary Medicine and Science, University of Nottingham, Sutton Bonington, United Kingdom

**Keywords:** PDAC, INSL3, immunoassay, biomarker, pancreatic cancer

## Abstract

Pancreatic ductal carcinoma (PDAC) is a rapidly growing cancer with a very poor prognosis. It is, therefore, important to develop novel, specific biomarkers to identify such cancers as early as possible. In a recent article published in *Nature Cell Biology*, Yeom and colleagues postulated that circulating insulin-like peptide 3 (INSL3) might serve as such a biomarker. Experiments were first conducted in *Drosophila* to show that the Dilp8/Lgr3 system regulated the fly equivalent of cachexia. This was then translated to humans to imply the involvement of the INSL3/RXFP2 system in PDAC-associated cachexia and that circulating INSL3 might serve as an early PDAC biomarker. We have now analyzed blood and tumor tissue from PDAC patients using a well-validated and recognized INSL3 immunoassay and specific antihuman INSL3 antibodies, and find no evidence to support these claims. We consider that this is largely due to Yeom and colleagues using a poorly validated immunoassay and antibodies for INSL3. Unfortunately, therefore, this peptide is not suitable for consideration as a PDAC biomarker.

## Background

Recent projections suggest that the worldwide incidence of pancreatic cancer will rise from 1.4 million new cases in 2020 to 2.9 million by 2040 (1). Pancreatic ductal adenocarcinoma (PDAC) patients do not display distinct early symptoms of the disease, nor are there national serum-based screening programmes in place. As a result, approximately 80% of patients are diagnosed to have locally advanced or metastatic disease at the time of presentation. This results in a low surgical resection rate, and many patients die within one year of diagnosis. Early detection of PDAC with a reliable biomarker would enable earlier intervention, potentially leading to improved survival rates. So far, global proteomic approaches have failed to identify suitable biomarkers.

However, in a recent study using a *Drosophila* model of cancer anorexia, it was found that a tumor-derived Dilp8-LGR3 system within brain neurons might be responsible for the associated muscle wasting, typical in humans of cancer-associated cachexia (2). Though evolutionarily far removed, it is suggested that the human equivalent to this hormone-receptor system might be represented by the insulin-like peptide hormone, insulin-like peptide 3 (INSL3), and its specific receptor RXFP2 (2, 3). Furthermore, Yeom and colleagues (2), using mouse cancer models as well as findings from human cancer patients, postulated that INSL3 was produced by tumors, such as PDAC, and was responsible for inducing cachexia in these patients. In particular, they showed that intracerebroventricular, though not intraperitoneal, injection of INSL3 in mice reduced food intake; that some, though not all, pancreatic cancer cell lines expressed and secreted INSL3 peptide into culture media; and that circulating INSL3 was higher in blood from PDAC patients, especially those suffering cachexia, and thus might be considered a new cancer biomarker (2). This was further supported by immunohistochemical identification of INSL3 staining in PDAC cells, though not in normal pancreas (2).

While not wishing to detract from the *Drosophila* studies, we note that almost all the human-related findings made use of a single immunoassay for INSL3, for which no validation evidence is provided, and where the results suggest erroneous findings. We have been developing and reviewing INSL3 immunoassays for many years; currently, only three appear to meet appropriate validation criteria (4). Using one of these assays, we have reassessed here the role of INSL3 as a PDAC biomarker and its possible role in cancer cachexia.

## Materials and methods

### INSL3 immunoassay

INSL3 was measured in cell culture media and in venous serum using a well-validated time-resolved fluorescence immunoassay (TRFIA) (5, 6), which yields results very similar to other established and validated assays, including a new LC/MS-MS-based assay (7). The limit of detection for the assay version used here is 20 pg/ml, with intra- and interplate coefficients of variation of < 3% and < 9%, respectively. There is no cross-reactivity with any other insulin-like molecules (e.g., insulin, relaxin, IGF1, IGF2), nor other proteins as far as is known. We have shown elsewhere that INSL3 in serum is stable over several years and after multiple (up to 5) freeze–thaw cycles. For comparative purposes (see **Supplementary Figure S1**), another INSL3 immunoassay was purchased from MyBiosource (No. MBS2024491; San Diego, CA, USA), presumably similar to the one used by Yeom and colleagues (2) and used on duplicate male and female human serum samples exactly following the manufacturer’s procedure.

Serum samples from patients with pancreatic cancers were obtained before and after surgical resection at the University Medical Center Schleswig-Holstein (UKSH), following written consent and approval by the ethics committee of the University of Lübeck (No. 16-281). Histological diagnosis of each specimen was confirmed by the final pathology report (**Table 1**).

**Table 1:** Circulating INSL3 concentration pre- and postpancreatic tumor resection.

No.	Tumor type	Sex	Age	BMI	INSL3 pre	INSL3 post	Δ INSL3
1	ASC	F	73	27.1	0.227	0.201	0.026
2	NET	F	75	24.8	0.166	0.181	− 0.015
3	PDAC	F	75	33.5	0.156	0.245	− 0.089
4	PDAC	F	59	18.6	0.274	0.354	− 0.08
5	PDAC	F	69	24.9	0.270	0.433	− 0.164
6	IPMN	M	55	26.7	1.274	1.519	− 0.245
7	NET	M	55	30.1	0.902	0.903	− 0.001
8	PDAC	M	75	25.6	1.740	2.262	− 0.521
9	RCC metastases	M	78	24.6	1.297	0.979	0.318
10	DASC	M	61	18.3	2.057	1.804	0.253
11	NET	M	48	28.4	1.359	1.347	0.012
12	PDAC	M	79	29.6	0.481	0.261	0.220
13	PDAC	M	55	27.1	2.067	1.549	0.518
14	SCA	M	71	31.2	1.261	1.444	− 0.183
15	IPMN	M	77	27.4	1.244	0.864	0.379
16	PDAC	M	76	26.9	0.249	0.383	− 0.134

*ASC*, adenosquamous carcinoma; *NET*, neuroendocrine tumor; *PDAC*, pancreatic ductal adenocarcinoma; *IPMN*, intraductal papillary mucinous neoplasm; *RCC*, pancreatic metastasis of renal clear cell carcinoma; *DASC*, ductal adenosquamous carcinoma; *SCA*, serous cystic adenoma; *BMI*, body mass index; *D-INSL3*, difference in INSL3 concentration between pre- and post- tumor resection.

### Cell and tissue culture

PDAC cell lines were obtained from ATCC (LGC Standards, Teddington, UK), and all culture media and biochemicals were sourced from Gibco–Life Technologies (Thermo-Scientific, Loughborough, UK) or Sigma-Aldrich (Merck, Gillingham, UK). CAPAN-1 cells (HTB-79TM) have an epithelial morphology and were isolated from the pancreas of a 40-year-old white man with pancreatic adenocarcinoma. PANC-1 cells (CRL-1469TM) were similarly isolated from the pancreatic duct of a 56-year-old Caucasian man with epithelioid carcinoma. The human HPDE6c7 cell line was obtained directly from benign pancreatic ductal epithelial cells by immortalization (Merck Life Science, Gillingham, UK). CAPAN-1 cells were cultured in Iscove’s modified Dulbecco’s medium containing 1% l-glutamine, 1% penicillin, and 10% fetal bovine serum (FBS). PANC-1 cells were cultured in DMEM:F12 containing 1% l-glutamine, 1% penicillin, and 10% FBS. HPDE cells were cultured in keratinocyte serum-free medium (K-SFM) basic medium containing 0.2% K-SFM growth supplement and 0.05% gentamicin. Fresh PDAC tissue specimens from patients undergoing pancreatic resection at our department were cultured as organotypic slice cultures (OTSCs) after written consent and ethical approval from the ethics committee of the University of Lübeck (No. 16-281). OTSCs were cultivated as described previously (8). Tissues were either (i) untreated or treated with (ii) gemcitabine (89.3 µM)/paclitaxel (4.27 µM) or (iii) folfirinox (5-FU: 426 µM; oxaliplatin: 4.96 µM; irinotecan: 5.78 µM). Tissue culture supernatants were collected every 24 h and frozen at − 80°C until further analysis.

### RT-PCR for INSL3 and JAK3

RNA was extracted from cultured cells at 24 and 48 h using the Qiagen RNase^®^ Plus Mini Kit, which includes gDNA eliminator treatment. A total of 2 µg RNA was used to synthesize cDNA using the Takara PrimeScriptTM IV First Strand cDNA Synthesis Mix Kit (Takara Bio, Saint-Germain-en-Laye, France). RT-PCR was carried out for human INSL3 (forward primer: cctggtgttcgcgttggg; reverse primer: cagagggtcagcaggtcttg) and human JAK3 (forward primer: ccactccctctttgctctgg; reverse primer: caccctgctccttgagactg) gene transcripts using a Rotor-Gene 3000 (Qiagen; Manchester, UK). DNA was denatured at 95°C for 45 s; annealing was carried out at 64°C, and elongation at 72°C, each for 1 min. As a control for the integrity of the RNA, all samples were also validated for the expression of the human housekeeping gene *RPS27a* (forward primer: ccaggataaggaaggaattcctcctg; reverse primer: ccagcaccacattcatcagaagg), with a cycling program for 20 cycles at 95°C for 20 s, annealing at 64°C for 20 s, and elongation at 72°C for 30 s. All primers were synthesized by Eurofins Genomics (Ebersberg, Germany). Each reaction mixture of 20 µl contained 1 µl of the cDNA, 1 µM of each primer, and 1× TaKaRa TB Green^®^ Premix (Takara Bio Europe, St-Germain-en-Laye, France). PCR products were checked for size and integrity on 1.5% agarose gels. All PCR products were analyzed by gel electrophoresis on 1.5% agarose gels containing 1% GelRed^®^ Nucleic Acid Stain (Biotium; Cambridge Bioscience, Cambridge, UK), followed by fluorescence digitization using the Chemidoc Imaging System (Bio-Rad, Watford, UK).

### Immunohistochemistry and tissue microarrays

Immunohistochemistry (IHC) for human INSL3 was carried out by Source BioScience Histopathology (Nottingham, UK) on formalin-fixed, paraffin-embedded 4-mm sections of human testis and similarly prepared sections of human pancreatic tumor tissue assembled in the form of a tissue microarray (TMA) (9). IHC for all tissues was carried out in parallel after trials to optimize the concentration of the primary anti-INSL3 antibody (1:2,500; HPA028615, Atlas Antibodies AB, Stockholm, Sweden). This antibody has been well characterized and is highly specific for human INSL3 (10). Specific anti-INSL3 binding was visualized using a conventional horseradish peroxidase-bound secondary antibody and DAB. Sections were then lightly counterstained with hematoxylin. Negative controls for testis omitted the primary antiserum, while positive controls for the TMA used a well-characterized anti-Ki67 (30-9) rabbit monoclonal antibody (Roche Diagnostics, Burgess Hill, UK) to demonstrate accessibility of antibodies to the tissues under the conditions used.

## Results and discussion

### Circulating INSL3 levels in patients with pancreatic cancer

Firstly, in our study at entry, INSL3 levels (mean ± SD; 1.27 ng/ml ± 0.57 ng/ml) in the men corresponded well to the reference levels estimated for community-dwelling men in this age group (4, 7, 11). These were significantly different from the comparable values for women (mean ± SD; 0.22 ng/ml ± 0.05 ng/ml). There are currently no reference values for postmenopausal women, though mostly sporadic testing in women has indicated values close to the assay level of detection (5–7). Whereas in men, INSL3 derives uniquely from the permanent population of adult-type Leydig cells in the testes, in women, low circulating INSL3 derives largely from the equivalent theca interna cells of growing antral follicles in the ovaries of women of reproductive age (6), which are mostly absent after the menopause. It is to be noted that in the article by Yeom and colleagues (2), circulating INSL3 appeared to be similar in men and women, with the mean and range in the men consistently below those of the normal population (as measured by others), even in the control (benign disease) groups.

The INSL3 immunoassay used by Yeom and colleagues has also recently been used in another study, which likewise produced erroneous results (12). In that study, circulating serum levels of INSL3 in women of reproductive age were reported to range between 1,500 and 2,000 pg/ml, with lower levels reported in follicular fluid (12). In fact, serum levels of INSL3 in women or monkeys, as measured by two separate and well-validated INSL3 assays, are typically well below 500 pg/ml during the follicular phase (6, 13, 14), whereas follicular fluid concentrations, close to the theca cell source of INSL3, are up to 10-fold higher than in serum (13, 14). In **Supplementary Figure S1**, we present a direct comparison between the well-validated TRFIA INSL3 assay and the assay used by Yeom and colleagues. It is evident that the latter assay is measuring something other than INSL3, or in addition to it, and thus cannot deliver reliable results for this peptide.

**Figure 1:**
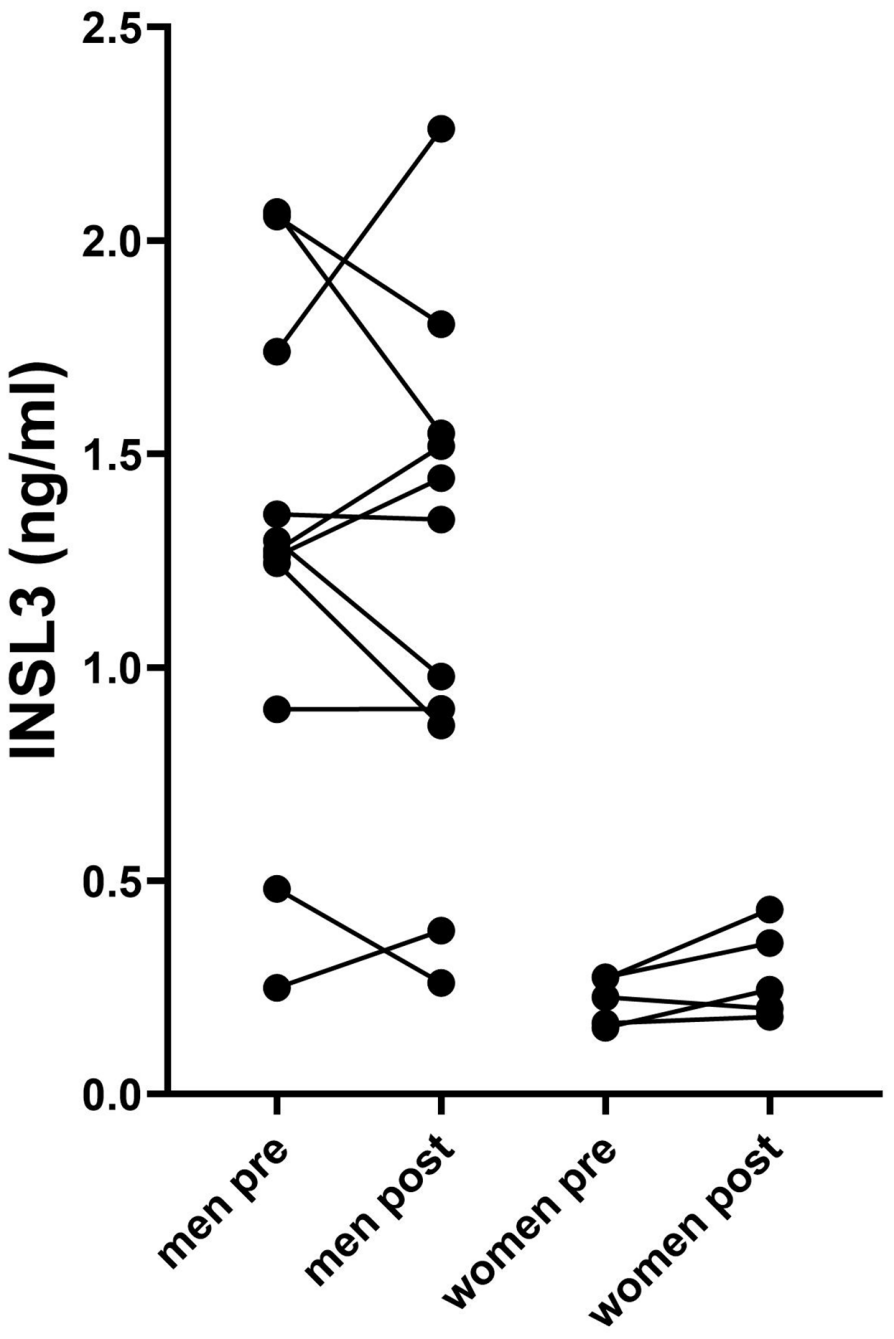
Circulating INSL3 concentrations in men and women pre- and post- resection of pancreatic tumor tissue. Lines connect individual patients. Data were log-transformed prior to the *t*-test comparison.

We also measured circulating INSL3 in patients before and after surgical resection of pancreatic cancers (**Figure 1**; **Table 1**). There was no evidence of any significant lowering of the circulating INSL3, as would be expected if the tumorous tissue were a source of the circulating peptide. Even for the PDAC patients, some showed a small decrease, some a small increase, without an obvious pattern. Similarly, for the anorexic patients (BMI < 20), no clear pattern of INSL3 reduction was evident.

**Table 1** includes data from different kinds of pancreatic cancers, including PDAC and neuroendocrine carcinomas, which are known to have a range of pathologies and outcomes (15). Nevertheless, INSL3 values did not indicate any significant relationship to any particular cancer type and were essentially not different from noncancer subjects.

### Pancreatic cancer cell culture

**Figure 2:**
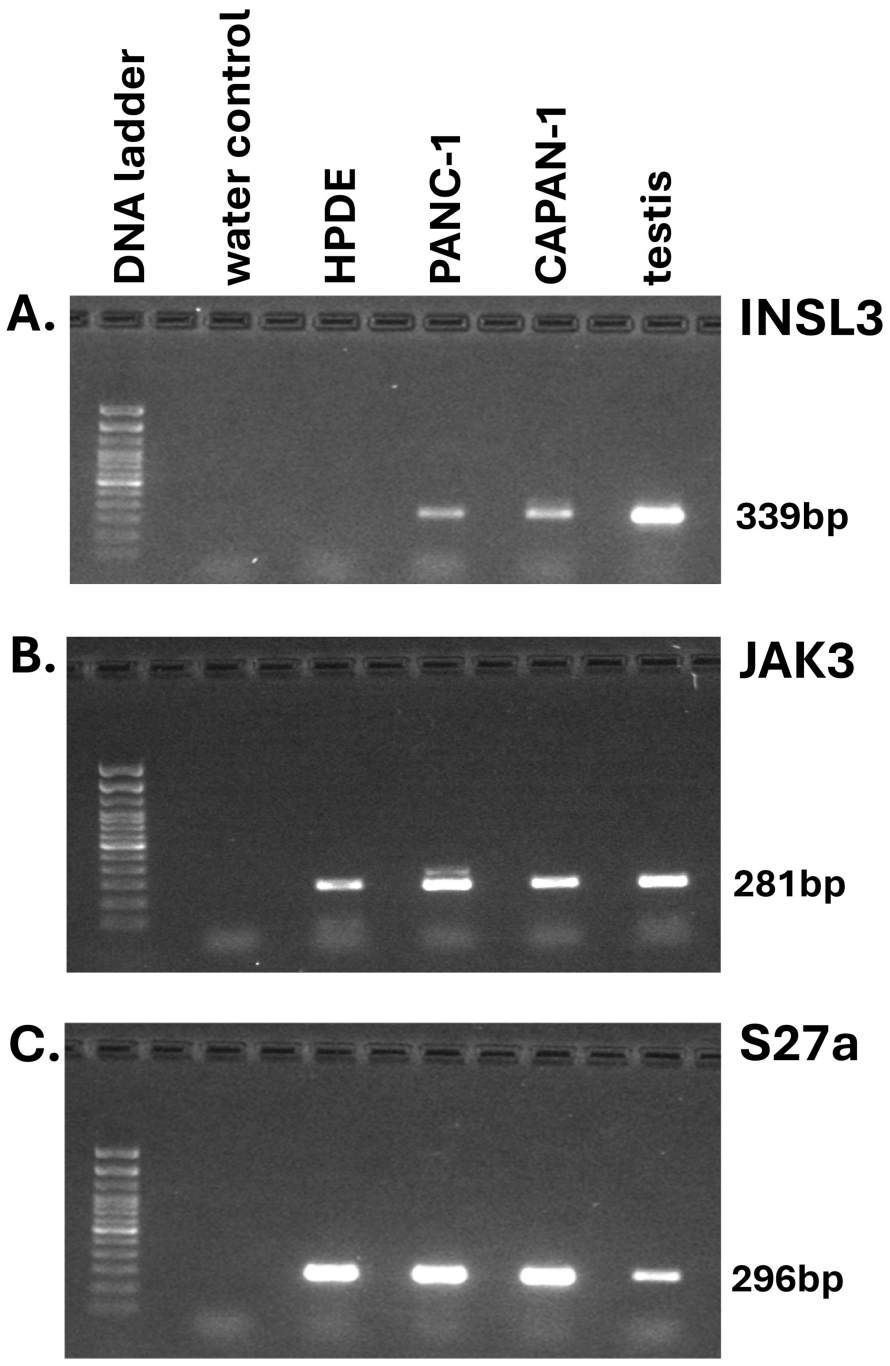
RT-PCR analysis by gel electrophoresis of final PCR products for INSL3 (insulin-like peptide 3; **(A)** and JAK3 (janus kinase 3; **(B)** of RNA derived from pancreatic adenocarcinoma (PANC-1, CAPAN-1) and immortalized pancreatic epithelial (HPDE) cell-lines. Transcripts for the small ribosomal protein S27a **(C)** served as housekeeping control.

Several different malignant and benign pancreatic cell lines are available from ATCC or other sources. PANC-1 (PDAC, quasi-mesenchymal subtype), CAPAN-1 (PDAC, epithelial subtype), and HPDE cells (normal pancreatic ducts) were cultured for up to 96 h in appropriate media (initial density ca 4 × 10^5^ cells per well of six-well plates). While all adenocarcinoma-derived cell lines indeed evidenced expression of INSL3 mRNA by RT-PCR (**Figure 2**), none indicated the secretion of any INSL3 peptide into the culture medium (experiments carried out at least four times with independent triplicates and using different passage numbers; not shown), in contrast to the results presented by Yeom and colleagues using their immunoassay (2). Similarly, replicate independent primary PDAC OTSC were established from fresh tumor tissue and grown for up to 72 h, either treated or not with clinically relevant standard treatments (i.e., folfirinox or gemcitabine/paclitaxel). In no case could INSL3 peptide be detected in the culture media (not shown).

The *INSL3* gene on human chromosome 19 is located only about 3 kb upstream of the last exon of the *JAK3* gene, and attention has been drawn to the finding that, in diverse other tumor cell types, there may be expression of a JAK3-INSL3 fusion transcript where read-through RNA is transcribed from an actively expressed *JAK3* gene (16, 17). This does not imply an intention to express functional INSL3 transcripts, but merely that isolators between the two genes are not operational when the upstream gene, in this case JAK3, acts as a strong driver. In such a context, evidence by RT-PCR of INSL3 transcripts is not evidence of INSL3 peptide synthesis. JAK3 mRNA expression was noted in all the PDAC cell lines tested (**Figure 2**).

**Figure 3:**
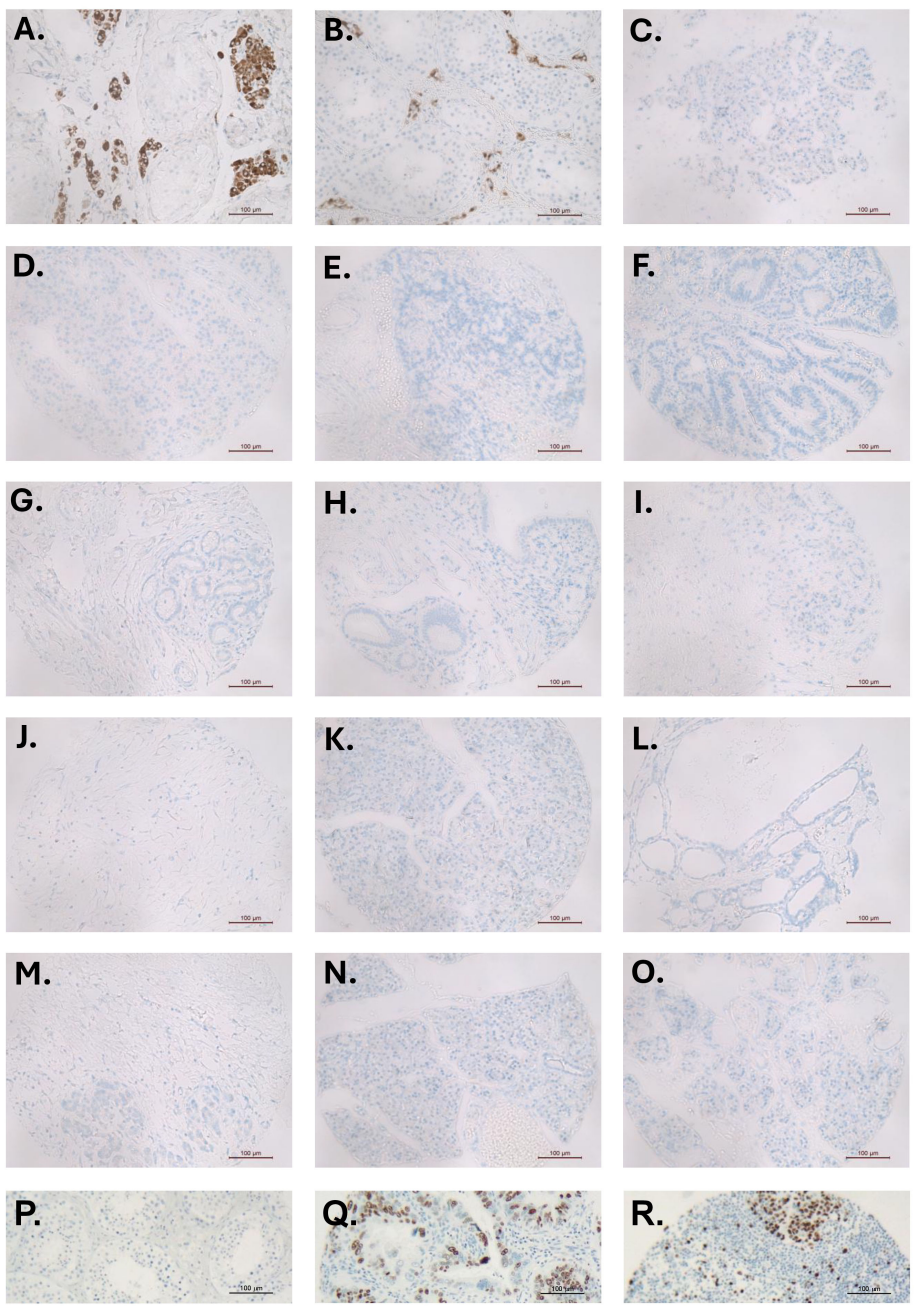
Immunohistochemistry for INSL3 (brown staining) in sections of different human testes **(A**, **B)**, used as positive controls, and 13 different pancreatic tumor tissue microarray sections **(C–O)**. In **(A**, **B)**, only the Leydig cells—and no other cell type—are stained. There is no specific INSL3 staining in any of the pancreatic tumor TMA sections. **(P)** testis negative control; **(Q, R)** TMA positive controls showing nuclear Ki67 staining (brown).

Yeom and colleagues (2) further support their findings by presenting an immunohistochemical image of a single PDAC tissue section stained using an undefined and unvalidated INSL3 antibody, and without appropriate positive and negative controls. For those working in this field, such antibodies are notorious for presenting false-positive results; there are very few available antibodies that are able specifically to label human INSL3-producing cells, especially where production is modest. We have applied one such well-validated antibody (10) to a pancreatic cancer tissue array without any positive result (**Figures 3C–O**), other than in two independent testis control sections (**Figures 3A, B**), in which only the Leydig cells, and no other cell type, are immunostained.

## Conclusion

It seems logical to extrapolate the interesting results from the *Drosophila* cancer-anorexia model involving the Dilp8-LGR3 system to pancreatic cancer in humans. However, this translation in the original study was flawed by the mistaken use of a poorly validated INSL3 immunoassay and other antibodies, which, unfortunately, appeared to corroborate this hypothesis. Our present study shows that INSL3 peptide is not significantly produced by the tumor cells themselves or by any cells within PDAC tissue, and thus cannot be involved in a mechanism to induce cachexia. Moreover, there is, as yet, no mechanism offered by which INSL3 in the circulation might be able to cross the blood–brain barrier to influence RXFP2 receptors in the relevant brain areas. This was, in fact, demonstrated by Yeom and colleagues, who found no influence of intraperitoneal INSL3 on cachexia in their mouse model, unlike intracerebroventricular INSL3 (2). We cannot, therefore, support the notion of INSL3 as a novel pancreatic cancer biomarker.

## Data Availability

The raw data supporting the conclusions of this article will be made available by the authors, without undue reservation.

## In Memoriam

Professor Hendrik Ungefroren sadly passed away after completion of this study. We dedicate this article to him.
