# Supplementary material for: Insulin-like peptide 3 is not a biomarker for pancreatic ductal adenocarcinoma
Source: Front Endocrinol (Lausanne). 2025 Aug 27;16:1625906. doi: 10.3389/fendo.2025.1625906 (PMC12420327; doi:10.3389/fendo.2025.1625906)
Supplement: Supplementary file 1 [file DataSheet1.docx]

**Supplement Figure 1**

**Direct comparision of INSL3 TRFIA assay with the MyBiosource INSL3 ELISA**.

**
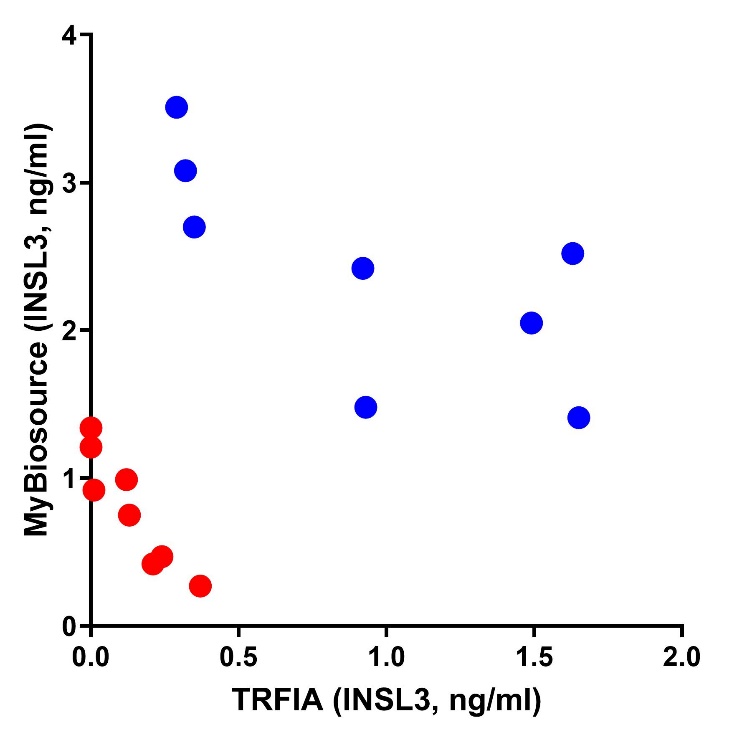
 INSL3 concentration (ng/ml)**

| **Sex** | **TRFIA** | **MyBiosource** |
| --- | --- | --- |
| *male*  *female* | 0.29  0.32  0.35  0.92  0.93  1.63  1.65  1.49  0  0  0.01  0.13  0.12  0.21  0.37  0.24 | 3.51  3.08  2.70  2.42  1.48  2.52  1.41  2.05  1.34  1.21  0.92  0.75  0.99  0.42  0.27  0.47 |

Female (n=8; red circles) and male (n=8; blue circles) serum samples were selected from frozen archived material (stored at -80^o^C) to offer a range of INSL3 concentrations. These were measured in parallel using the established, well-validated TRFIA assay [5] and the MyBiosource ELISA [2,12] exactly following the manufacturer’s instructions (values averaged from duplicates; <10% variation between duplicates). There is a complete lack of correlation between the assays.
